# Diagnosis and Treatment of Primary Cutaneous B-Cell Lymphomas: State of the Art and Perspectives

**DOI:** 10.3390/cancers12061497

**Published:** 2020-06-08

**Authors:** Maëlle Dumont, Maxime Battistella, Caroline Ram-Wolff, Martine Bagot, Adèle de Masson

**Affiliations:** 1 Department of Dermatology, APHP, Saint-Louis Hospital, F-75010 Paris, France; maelledumont@gmail.com (M.D.); caroline.ram-wolff@aphp.fr (C.R.-W.); adele.demasson@aphp.fr (A.d.M.); 2 INSERM U976, Human Immunology, Pathophysiology and Immunotherapy, Institut de Recherche Saint-Louis, F-75010 Paris, France; maxime.battistella@aphp.fr; 3 Faculty of Medicine, Université de Paris (Paris University), F-75010 Paris, France; 4 Pathology, APHP, Saint-Louis Hospital, F-75010 Paris, France

**Keywords:** cutaneous B-cell lymphomas, lymphoid malignancies, B-cells, lymphocytes, skin, lymphomas, B-cell lymphomas, review

## Abstract

Primary cutaneous B-cell lymphomas are rare entities that develop primarily in the skin. They constitute a heterogeneous group that represents around a quarter of primary cutaneous lymphomas. The 2018 update of the World Health Organization-European Organization for Research and Treatment of Cancer (WHO-EORTC) classification differentiates primary cutaneous marginal zone lymphoma and primary cutaneous follicle center lymphoma with an indolent course from primary cutaneous diffuse large B-cell lymphoma, leg type with an aggressive behavior. The broad spectrum of clinical presentations and the disease course marked by frequent relapses are diagnostic and therapeutic challenges. The classification of these diseases has been refined in recent years, which allows to better define their immunopathogenesis and specific management. In the present article, we review the main clinico-biological characteristics and the current therapeutic options of these three main subsets. Based on the recent therapeutic advances in nodal B-cell lymphomas, we focus on the development of novel treatment options applicable to primary cutaneous B-cell lymphomas, including targeted therapies, combination treatments and immunotherapeutic approaches, and cover basic, translational and clinical aspects aiming to improve the treatment of cutaneous B-cell lymphomas.

## 1. Introduction

Primary cutaneous B-cell lymphomas (PCBCL) represent approximately 20 to 25% of all primary cutaneous lymphomas (PCL) [1,2]. The incidence of these rare entities is estimated to be <1 per 100,000 people/year and increases with age [1,2,3].

By definition, PCBCL are present in the skin with no evidence of extracutaneous disease at the time of diagnosis. They belong to the group of lymphoid malignancies, which are currently defined according to the 2016 revision of the WHO classification of lymphoid neoplasms [4]. The World Health Organization-European Organization for Research and Treatment of Cancer (WHO-EORTC) classification has recently been updated to best define this heterogeneous group of primary cutaneous lymphomas [1,2]. In the 2018 update of the WHO-EORTC classification [2], the three most common entities are primary cutaneous marginal zone lymphoma (PCMZL), primary cutaneous follicle center lymphoma (PCFCL) and primary cutaneous diffuse large B-cell lymphoma, leg type (PCDLBCL, LT). PCMZL and PCFCL have an indolent behavior while PCDLBCL, LT is an aggressive subset.

Intravascular large B-cell lymphoma (IVLBCL) is an extremely rare entity, most often associated with extracutaneous involvement (central nervous system, lung) that can also present with skin-limited disease. Included as a new provisional entity in the 2016 revision of the WHO-classification [4] and in the updated 2018 classification [2], EBV^+^ mucocutaneous ulcer (EBVMCU) is also very rare and defined as an ulceration of the skin, oropharyngeal mucosa, or gastrointestinal tract in immunocompromised patients (such as elderly patients and/or patients treated with methotrexate, cyclosporine, azathioprine, or tumor necrosis factor alpha inhibitors).

The staging of PCBCL has been defined by the WHO/EORTC [5] and a CT scan at least is recommended at baseline to rule out systemic involvement.

Optimal management of PCBCL requires multi-disciplinary collaboration between dermatologists, hematologists, pathologists and radiation oncologists. Guidelines for the treatment of PCBCL have been published by the EORTC [6].

This review describes the epidemiological, clinical, histopathological, cytogenetic and molecular features of each of the three most frequent PCBCL subtypes and focuses on the current therapeutic options and future developments in the management of PCBCL.

## 2. Indolent PCBCL

### 2.1. Primary Cutaneous Marginal Zone Lymphoma

#### 2.1.1. Epidemiology/Prognosis

PCMZL accounts for 9% of the PCL and is the second most common PCBCL. The five-year survival rate is around 99% [2]. It typically affects medium-aged adults although pediatric cases have also been reported [7].

#### 2.1.2. Diagnosis

**Figure 1:**
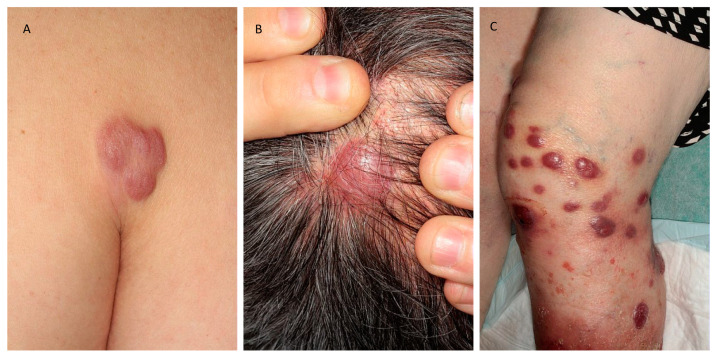
Clinical presentations of the three main subsets of primary cutaneous B-cell lymphomas. (**A**) Primary cutaneous marginal zone lymphoma; (**B**) primary cutaneous follicle center lymphoma; (**C**) primary cutaneous diffuse large B-cell lymphoma.

PCMZL usually presents with erythematous to violaceous papules, plaques, nodules or tumors (Figure 1A), sometimes infiltrated but ulceration is atypical. Peri-lesional annular or diffuse erythema is possible [8]. Solitary or multifocal, the lesions are localized preferentially on the trunk or the upper extremities. The lesions can regress spontaneously and rarely give way to anetoderma [9]. PCMZL manifesting as AL amyloidoma of the skin, without systemic amyloidosis, has also been reported [10]. Cutaneous relapses occur in half of the cases but extracutaneous spread is very uncommon [11], as well as transformation to high-grade lymphoma [12].

#### 2.1.3. Histology

**Figure 2:**
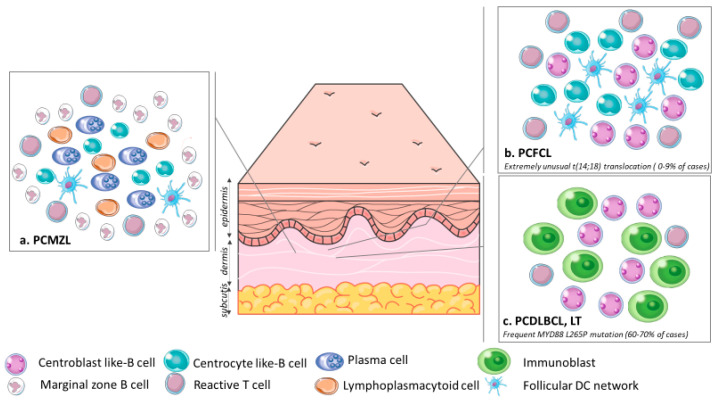
Organization of the tumor cells and microenvironment in (**a**). PCMZL: Infiltrate made of small centrocyte-like B-cells, lymphoplasmacytoid cells, plasma cells and reactive T cells admixed with a FDC network surrounded by marginal zone B cells; (**b**). PCFCL: Infiltrate made of centrocytes and centroblasts often with a FDC network and scattered reactive T cells; (**c**). PCDLBCL, LT: A monomorphic population of large atypical cells ressembling centroblasts and immunoblasts. The t (14; 18) translocation is extremely rare in PCFCL, unlike in primary nodal follicular lymphoma. The PCDLBCL, LT is characterized by frequent MYD88 L265P mutations, which helps discriminate PCDLBCL, LT from PCFCL with large cells, in which the MYD88 L265P mutation is absent. Abbreviations: PCMZL, primary cutaneous marginal zone lymphoma; PCFCL, primary cutaneous follicle center lymphoma; PCDLBCL, LT, primary cutaneous diffuse large B-cell lymphoma, leg type; DC, dendritic cell.

The infiltrate is made of small lymphocytes, small centrocyte-like B-cells, lymphoplasmacytoid cells, plasma cells and also reactive T cells (Figure 2a). Eosinophils are observed in about 25% of the cases [13]. Scattered follicles with reactive germinal centers (GC) are surrounded by marginal zone B cells with irregular nucleus, inconspicuous nucleoli and abundant pale cytoplasm [1].

#### 2.1.4. Immunohistochemistry, Cytogenetic and Molecular Features

Two types of PCMZL have been described [14,15]. Most PCMZL express class-switched immunoglobulins (IgG and more rarely IgA or IgE) with a predominance of T cells in a T-cell helper type 2 environment. Several cases with IgG4 expression have been reported [16,17]. The second subset of PCMZL shows a diffuse proliferation or large nodules of B cells expressing IgM and often CXCR3 [2].

Neoplastic cells are CD20^+^, CD79a^+^ and Bcl-2^+^ and negative for Bcl-6 and CD10 [8]. Plasma cells express CD138 and CD79a and have a monotypic expression of the kappa or lambda light chain. Immunophenotyping helps distinguish the class-switched immunoglobulins and the IgM-positive subset. Cases with class-switched immunoglobulins often have more prominent CD21^+^ follicular dendritic cell (FDC) networks and lack CXCR3 expression, unlike IgM-positive cases [14].

Recurrent alterations of the FAS gene have been recently highlighted, suggesting that a downregulation of apoptosis could be a mechanism explaining the indolent behavior of PCMZL [18].

Chromosomal abnormalities are uncommon in PCMZL. Rare t (14;18), t (3;14), t (11;18) or trisomy 3 or 18 have been reported, but if present, a systemic entity should be closely worked-up [19].

#### 2.1.5. Etiology

Etiology remains unknown. The main hypothesis is a chronic antigen stimulation. Different associations have been reported to date: *Borrelia burgdorferi* in European cases [20], *Helicobacter pylori* colonization of the stomach [21], *influenza* or viral hepatitis A vaccination, arthropod bites, traumatic injuries, tattoos [22,23,24]. Associations with gastrointestinal disorders and autoimmune diseases have been reported in PCMZL [24].

### 2.2. Primary Cutaneous Follicle Center Lymphoma

#### 2.2.1. Epidemiology/Prognosis

PCFCL are the most common PCBCL, representing about 60% of the cases. The five-year survival rate is around 95% although localization on the legs has been associated with a poorer prognosis. PCFCL typically affects middle-aged adults. Relapses occur in roughly half of the cases but extracutaneous dissemination is rare [2,3,25].

#### 2.2.2. Diagnosis

Clinical features are firm erythematous to purple macules, papules, plaques or tumors (Figure 1B). Often solitary, the lesions are mainly localized on the head and the trunk. Spontaneous regression is rare, and the lesions tend to increase in size without treatment. Some cases present as alopecic patches of the scalp [26].

#### 2.2.3. Histology

The neoplastic infiltrate presents with a follicular, follicular and diffuse, or diffuse growth pattern. The epidermis is spared with a grenz zone. Tumor cells are made of centrocytes, most often with small nuclei, and centroblasts. An FDC network and reactive T cells are often seen (Figure 2b). There are variants with large centrocytic cells with a clear cytoplasm, cases with spindle tumor cells, and also cases with Hodgkin and Reed Sternberg like cells [27,28,29]. The staging system used for nodal follicular lymphoma does not have prognostic value in PCFCL [30].

#### 2.2.4. Immunohistochemistry, Cytogenetic and Molecular Features

Neoplastic B cells are CD19^+^, CD20^+^, CD79a^+^, PAX5^+^, IgM^−^, Bcl-6^+^ and most often bcl-2^−^. Coexpression of Bcl-2 and CD10 should lead to rule out a primary nodal follicular lymphoma with secondary skin involvement [31,32]. Post germinal center (GC) markers IRF4/MUM1 and FOXP1 are negative, unlike in DLBCL, LT. The residual FDC network expresses CD21 and CD23.

The *MYD88* L265P mutation is absent (helping the distinction between DLBCL, LT and PCFCL with large cells) and the t (14;18) translocation is extremely rare (unlike in primary nodal follicular lymphoma).

### 2.3. Treatment of Indolent Lymphoma

In localized disease, first-line therapies are usually local radiation or surgical excision. The effectiveness of antibiotics in the case of a *Borrelia burgdorferi* positive serology is still controversial [22,33,34]. A wait-and-see attitude is considered possible in the EORTC [6] and the National Comprehensive Cancer Network (NCCN) guidelines [35]. The expectant management could be distressing for patients with PCBCL given the impact of the disease on health-related quality of life (HRQoL) [36].

Complete response (CR) rate is close to 100% with local radiation, although relapses and acute adverse events (AE) occur in almost half of the cases [37]. Retrospective cohorts help define the optimal dose to reduce toxicity and maintain a high response rate. No significant difference between very low dose (4–8 Gy) and standard dose (>24 Gy) has been found by Goyal et al. [38] but the response rate was significantly lower in the very low dose group (4 Gy) versus standard dose (>24 Gy, median 40 Gy) in a recent study on PCMZL and PCFCL [39]. The European Society for Medical Oncology (ESMO) guidelines recommend a standard dose of 24 to 30 Gy for localized disease and a low dose of 4 Gy for the palliative treatment of disseminated disease [40].

A retrospective study on indolent PCBCL found no significant difference in terms of five-year disease free survival (DFS, 96%) between high-dose (30 to 40 Gy) radiation and surgery after an average follow-up of 3.6 years [41].

Disseminated lesions may be treated with intravenous rituximab, an anti-CD20 monoclonal antibody. According to a literature review by Morales et al., durations of response ranged from 4 to 39 months with a median of 14 months for PCFCL and 9 months for PCMZL. CR rate was higher for PCFCL (77%) than for PCMZL (43%) [42]. However, patients were treated with variable numbers of infusions.

When lesions are difficult to treat by radiotherapy or surgery, such as the face and the scalp, treatment with intralesional rituximab has been considered. Indeed, the CR rate ranged from 83% to 89% [6]. In a recent study, the favorable clinical outcome was correlated with AEs such as marked flu-like symptoms [43]. In a small retrospective study, Kerl et al. observed that in two-thirds of patients with relapses, new lesions occurred at another site [44]. There was no recurrence on the injected lesions. Response rates were quite similar between PCMZL and PCFCL treated with intralesional rituximab [44,45].

In disseminated disease, another option is subcutaneous interferon-alpha. In a retrospective study, the overall response rate (ORR) was 66.7% with a median duration of response of 15.5 months, but the relapse rate was around 90% after 40 months of median follow-up [46].

Interferon-alpha (IFN-α) has also been used intralesionally in PCMZL. Eight patients received intralesional injections of three million IU, three times per week. All patients reached a CR after a median of eight weeks, two patients relapsed locally, and experienced a secondary CR after treatment with IFN-α. No extracutaneous relapses were reported and side effects were generally mild [47].

To increase the efficacy of immunotherapy, antibodies have been combined with radioisotopes. Yttrium-90 ibritumomab tiuxetan (IbT) is an anti-CD20-antibody conjugated with an isotope that delivers the cytotoxic effects of radiation to CD20-expressing B cells. In a retrospective cohort of 10 patients including 8 PCFCL and 2 PCDLBCL, LT, the ORR was 100% with a median time to relapse of 12 months [48]. IbT was approved by the Food and Drug Administration (FDA) for systemic B-cell lymphomas (BCLs) but has not been further explored in PCBCL. Despite this efficacy, application of IbT has been limited by concerns about treatment-related myeloid neoplasms (including myelodysplastic syndrome and acute myeloid leukemia) as a consequence of diffuse irradiation of the bone marrow [49].

Recently rituximab has also been combined with lenalidomide. A phase III study of lenalidomide plus rituximab versus placebo plus rituximab in relapsed or refractory indolent lymphomas showed encouraging results with a significantly increased PFS (hazard ratio of 0.46, 95% CI, 0.34 to 0.62) [50].

Other immunotherapeutic approaches include intralesional adenovirus-interferon gamma (TG1042), a non-replicating human adenovirus vector allowing the intracellular transfer of human interferon (IFN)-gamma cDNA. IFN-gamma gene delivery induces an immune response to lymphoma tumor-antigens resulting in the regression of injected but also noninjected lesions. A phase 1 study on nine patients showed three CR and two partial responses (PR) [51]. Three cases of multilesional CBCL among 21 patients were treated in a phase 2 clinical trial: one local CR, one local PR and one global response with disappearance of noninjected distant lesions were observed [52]. The results of the last clinical trial on relapsing PCBCL including six PCFCL, six PCMZL and one PCDLBCL other than leg type, were published in 2014. The ORR was 85% with a majority of CR. Median time to progression was 23.5 months. Side effects were mild to moderate with reactions at the injection site or flu-like syndromes [53].

Topical imiquimod monotherapy has been recently studied in CBCL in a retrospective monocentric cohort in the United States. Sixteen patients with indolent CBCL (T1aN0M0 to T3aN0M0) were treated with imiquimod 5% cream. The ORR was 62% including 31% of complete and 31% of partial responses with a median duration of treatment of 4.6 months (range 1.4 to 9.8 months). All CR occurred in T1a lesions. Histology type was not predictive of response. The only AE was local irritation. The relapse rate was not precise [54].

## 3. Aggressive PCBCL

Primary cutaneous diffuse large B-cell lymphoma, leg type

### 3.1. Epidemiology/Prognosis

PCDLBCL, LT represents 10–20% of PCL. It is an aggressive lymphoma with at least 10% of extracutaneous spread. Affecting mainly elderly women, the disease-specific-survival is around 56% [2,3].

### 3.2. Diagnosis

Lesions are generally localized on one or both legs, but other sites are involved in 10–15% of the cases. Painful, sometimes necrotic, the nodules or plaques rapidly grow into tumors that may be possibly ulcerated (Figure 1C). Solitary or more often multiple lesions can be observed [55,56].

### 3.3. Histology

The infiltrate is diffuse in the dermis and the subcutis. The epidermis is spared with a grenz zone, but ulceration is possible. A monomorphic population of large atypical cells resembling centroblasts in confluent sheets and sometimes immunoblasts with round cells are observed, mitotic figures are frequent (Figure 2c). Perivascular, reactive T cells are less common than in other PCBCL [57,58].

### 3.4. Immunohistochemistry, Cytogenetic and Molecular Features

B cell lineage markers help identify tumor cells: CD20^+^, CD79a^+^, PAX5^+^ with variable cytoplasmic IgM. Expression of Bcl-2 is strong, Bcl-6 variable and CD10 negative. In 75% of the cases, cMYC expression is present. When present, the double expression of MYC and Bcl-2 is evocative [59]. The expression of post GC markers IRF4/MUM1 and FOXP1 is also helpful for diagnosis. Proliferation is high with Ki67 around 60 to 90% [2,57,60]. Somatic mutations similar to the ABC (activated B cell)-type DLBCL have been observed by massive parallel sequencing [22,61]: mutations in the NFkB pathway such as the L265P *MYD88* mutation (60–70% of the cases), *TNFAIP3/A20*, *CD79B* and *CARD11* [62,63,64]. The *MYD88* mutation is a biomarker for diagnosis because it is absent in other primary cutaneous B-cell lymphomas, especially PCFCL with large centrocytes [59]. *cMYC* rearrangements and inactivation of *CDKN2a* by either deletion or promoter hypermethylation also participate in the pathophysiology of PCDLBCL, LT [64,65,66,67] and are associated with a poor prognosis [59,64,66]. More recently, Zhou et al. observed that 40% of 19 cases of PCDLBCL, LT had recurrent genetic alterations in PD-L1/PD-L2 [68].

### 3.5. Tumor Microenvironment

Mitteldorf et al. analyzed the tumor microenvironment in biopsy specimens of 16 PCDLBCL. They noticed 63% of CD33^+^ myeloid-derived suppressor cells (MDSCs) with PD-L1 coexpression but also immunosuppressive CD163^+^ M2 macrophages. Finally, in all the cases, tumor cells expressed membrane-bound PD-L1 suggesting a mechanism to escape immune surveillance [69].

Menguy et al. confirmed PD-L1 expression by numerous immune cells, characterized as CD163^+^ M2 macrophages, in the tumor microenvironment of PCDLBCL, LT [70]. On the other side, Felcht et al. found that CD4 and FOXP3 expression as well as the CD4/FOXP3 ratio were significantly decreased in PCLBCL, LT as compared to PCBCL of indolent behavior, suggesting that regulatory T cells may inhibit tumor progression in PCBCLs [71].

### 3.6. Treatment

PCDLBCL, LT is an aggressive lymphoma affecting elderly people and its management is thus challenging.

Local treatment is used as palliative treatment in patients unable to stand systemic treatments, or in combination with systemic treatments. Surgical excision of a single lesion, or local radiation therapy can help relieve the symptoms although the effect is mostly temporary [72].

Currently, first line treatment is rituximab and combination chemotherapy, most commonly cyclophosphamide, doxorubicine, vincristine and prednisone (CHOP)-like regimens if the general condition of the patient allows it [6,40]. In a large retrospective study evaluating survival and prognosis factors, Grange et al. showed that the combination of rituximab to polychemotherapy has significantly improved the prognosis of PCDLBCL, LT [56]. The prognosis may be further improved using age-adapted rituximab-polychemotherapy [73]. Based on favorable data of pegylated liposomal doxorubicine in primary cutaneous T cell lymphoma, five patients including four PCDLBCL, LT were treated in a phase II pilot clinical trial with good results. All the patients achieved CR after a median delay of three months, two relapsed. Side effects were generally mild, with only one case of grade 3 neutropenia. There was no need to decrease the doses or to interrupt the treatment [74]. A multicenter phase II clinical trial studied the association of pegylated liposomal doxorubicine and rituximab in 12 patients including three PCDLBCL, LT. Seven had relapsed disease, one-fourth had received radiotherapy as prior treatment. With a median follow-up of 56 months, the ORR of PCDLBCL, LT patients was 66% including 33% of complete and 33% of partial responses. The median time to best response was two months (ranging from one to four months) with a good safety profile: only grade 2 AEs were observed (neutropenia and palmar-plantar erythrodysesthesia) [75]. New combination treatments with rituximab need to be explored.

Rituximab combined with bendamustine, an intravenous alkylating agent, has been tried in DLBCL [76]. Retrospective data suggest that it may be a valuable treatment option in older patients [77]. A recent study reports the case of an 87-year-old woman with large nodules on the legs treated with rituximab plus bendamustine. Unfortunately the tumors progressed after two courses of treatment [78].

The preferential localization on the legs has led to the use of isolated limb perfusion with melphalan in a case report of a 61-year-old woman with relapsed disease. The response was complete and lasted for eight months with only mild lymphedema that resolved with regular compression massages [79].

PCDLBCL, LT have the phenotype and gene expression profile of ABC-type DLBCL and the ESMO guidelines recommend that they should be treated as other ABC-type DLBCLs [22,40,62]. Moreover, the presence of genetic abnormalities of the B-cell receptor pathway (such as *CD79B*) has been associated with a poor prognosis after first line treatment with R-CHOP [80] and could suggest the use of Bruton tyrosine kinase (BTK) inhibitors such as ibrutinib in the absence of associated *CARD11* or *PIM1* mutations that are associated with ibrutinib resistance [81,82]. BTK is an important signaling molecule of the B-cell receptor (BCR) signaling pathway. One study reported an excellent outcome with ibrutinib in a refractory PCDLBCL, LT after five therapeutic lines [83].

Multiple genetic abnormalities activating the NF-κB pathway support the use of lenalidomide, which targets the interferon regulatory factor (IRF)-4. In a multicenter, single-arm, phase II trial, Beylot-Barry et al. followed 19 patients with relapsing or refractory PCDLBCL, LT. The ORR was 26.3% and reduced doses tended to be associated with higher six-month ORR and progression-free survival (PFS). Five patients discontinued treatment because of hematologic, septic, cardiac or cutaneous toxicity. The *MYD88* mutation was not associated with a higher response rate, which is consistent with the fact that the *MYD88* mutation is associated with a poorer prognosis [59,64,84].

The discovery of a subset of PCDLBCL, LT with recurrent genetic alterations in PD-L1/PD-L2 [69] suggests that the use of immune checkpoint inhibitors could be explored. Di Raimondo reported the case of an 85-year-old man with a three-year history of DLBCL, LT refractory to three prior therapies. CR was observed with the association of rituximab, lenalidomide and pembrolizumab. Introduction of the treatments was sequential (rituximab first, then lenalidomide and finally pembrolizumab) with the aim to enhance the efficacy of the checkpoint inhibitor [85].

## 4. Therapeutic Perspectives in Indolent and Aggressive PCBCL

The use of novel agents developed in nodal BCL is being increasingly considered in PCBCL (Table 1).

### 4.1. Small Molecule Inhibitors

Since the discovery of the oncogenic activity of *MYD88* mutations, small molecules inhibitors have been used in DLBCL. A case report described the efficacy of ibrutinib in PCDLBCL, LT [83]. Next generation BTK inhibitors such as acalabrutinib and zanubrutinib are already used in other lymphomas [86,87,88].

The PI3 K/AKT pathway and mTOR signaling pathway play an important role in lymphomagenesis by transducing BCR activation signals [89]. mTOR inhibition with everolimus and temsirolimus, PI3 K inhibition with parsaclisib are rescue treatments of relapsed and refractory NHL [90] By targeting the PI3 K/AKT/mTOR cascade, these treatments reduce BCR signaling. A protein kinase C inhibitor, enzastaurin, that regulates the PI3 K, MAPK and JAK/STAT pathways in vitro, seems to act synergistically with ibrutinib in DLBCL [91,92].

Some Toll-Like-Receptors such as TLR7, TLR8 and TLR9 interact with MYD88. Immunomodulatory oligonucleotides (IMO) are used in immune-mediated inflammatory diseases [93,94]. IMO-8400, an antagonist of TLR7, TLR8 and TLR9 has been studied in patients with *MYD88(L265 P)*-positive DLBCL (NCT02252146).

Histone deacetylases (HDAC) are enzymes that interfere with immune surveillance. HDAC inhibitors can reverse immunosuppressive tumor environments. HDAC inhibitor monotherapy in B-cell lymphomas showed modest clinical benefit. The combination of HDAC inhibitor and immunotherapy could help overcome immunotherapy resistance by different mechanisms especially by modulating immune cell functions [95,96,97].

Although several subsets have strong Bcl-2 expression, venetoclax, a BCL2 inhibitor showed insufficient responses alone, but has been found to be a valuable ally in combination with chemo-immunotherapy [98,99,100,101].

### 4.2. Monoclonal Antibodies

Monoclonal antibodies have evolved very quickly over the last decades. Rituximab is often effective but the recurrence rate after treatment is still high in indolent and aggressive cutaneous lymphomas [6]. New generations of anti CD20 monoclonal antibodies (mABs) are designed to be more selective with less AEs and lower immunogenicity [102]. By binding to the small and the large loops of the CD20 cell surface antigen, ofatumumab improves complement-dependent cytotoxicity (CDC) and antibody-dependent cell-mediated cytotoxicity (ADCC). Ofatumumab has been used in several malignancies including DLBCL and FL [103,104,105].

MOR-208 is an Fc-enhanced, humanized anti-CD19 monoclonal antibody. Combination with lenalidomide showed an ORR of 58% as primary results of a phase II study in patients with relapsed or refractory DLBCL [106]. Dacetuzumab, a monoclonal anti-CD40 antibody reduced tumor bulk in a third of patients with B-cell non-Hodgkin lymphomas [107,108].

### 4.3. Antibody-Drug Conjugates

Antibody-drug conjugates (ADC) are monoclonal antibodies that bind specifically to tumor-associated target antigens and allow the delivery of highly potent cytotoxic agents.

Polatuzumab vedotin targets CD79 b using a CD79 b monoclonal antibody bound to vedotin, a toxic agent. This ADC has been studied combined with different treatments [109,110]. The CD79 protein is often expressed in PCBCL. Currently, POLARIX, a phase III trial, compares polatuzumab vedotin and R-CHOP with R-CHOP alone as initial treatment for DLBCL (NCT03274492). Targeting CD33, gemtuzumab ozogamicin is another ADC, which could be applied to PCDLBCL, LT. Expressed on most of the MDSCs, CD33 could be an interesting target in the tumor microenvironment [69,111].

### 4.4. Bi-Specific T-Cell Engaging (BiTE) Antibodies

Bi-specific T-cell engaging (BiTE) antibodies target a tumor antigen and an immune cell and lead to an antitumor immune response. Reactive T cells are often abundant in PCBCL. Binding to CD3 on T cells and CD19 on B cells, blinatumomab showed high response rates but was often associated with neurotoxicity [112].

Better tolerated, mosunetuzumab combines an anti-CD3 arm and an anti-CD20 arm. In a phase I/Ib trial, mosunetuzumab induces durable CRs with favorable tolerability in poor prognosis NHL including patients resistant to or relapsing after CAR-T therapy. A total of 218 patients were treated, mainly DLBCL (*n* = 87). ORR rate was 64.1% in indolent subsets, 34.7% in aggressive lymphomas and 43.8% if patients had received prior CAR-T therapy [113].

### 4.5. Fusion Proteins

DI-Leu16-IL2 is a recombinant fusion protein composed of the de-immunized and humanized anti-CD20 monoclonal antibody Leu16 and the human interleukin-2 (IL2). The anti-CD20 antibody binds to tumor B-cells, which may lead to antibody-dependent cell cytotoxicity, and the IL2 moiety stimulates natural killer and T-cell immune responses. DI-Leu16-IL2 has been used in a phase I study in melanoma [114] and is being assessed in BCLs including PCBCL (NCT00720135).

### 4.6. Tumor Vaccines

Considering the indolent course of PCMZL and PCFCL and their expression of a clonal BCR, vaccines designed to target tumor idiotypes have been developed [115]. Protein vaccines were evaluated as adjuvant therapy after systemic therapy in FL with promising results [116,117]. DNA vaccines are being studied in phase I trials (ISRCTN31090206) [118,119]. Cellular vaccines with DCs may induce durable tumor regression but need further investigations [120,121,122]. In situ vaccines offer the possibility to develop abscopal responses when combined with radiotherapy and TLR3 agonists (NCT01976585) [123].

### 4.7. Immune Checkpoint Inhibitors

Based on the better understanding of the microenvironment and mechanisms for escaping immune surveillance in lymphomas, immune checkpoint inhibitors have been tested with variable responses depending on the type of lymphoma. Combinations of treatments are increasingly explored. In a phase I trial, ipilimumab has been used in combination with rituximab in order to improve the efficacy of rituximab by increasing T-cell activation. Ipilimumab is a humanized IgG1 monoclonal antibody against CTLA-4, a co-inhibitory receptor expressed on T cells. Efficacy was modest in the entire cohort of 33 patients with relapsed or refractory BCLs with an ORR of 24%. However, the response was significantly better in follicular lymphoma patients with an ORR of 58% [124]. Combination of rituximab and ipilimumab should be further studied in indolent lymphomas.

### 4.8. Chimeric Antigen Receptor T Cells (CAR T Cells)

Adoptive cell therapies are currently explored in lymphomas. Among them, CAR-T cells are autologous T cells genetically modified to express a chimeric antigen receptor (CAR). The CAR recognizes tumor-specific antigens and leads to the killing of the neoplastic cells. To date in B cell NHL, two CD19-specific CAR-T cells axicel and tisagenlecleucel are FDA-approved to treat relapsed and refractory DLBCL after at least two lines of systemic therapy [125,126,127,128]. However, serious AEs (neurological side-effects and cytokine release syndrome) and resistance to CAR T-cells are important limits. Resistance may be partly due to the ability of tumor cells to defeat immunosurveillance mechanisms but also to a restricted distribution of the CAR T-cells [129]. Combination with immune checkpoint inhibitors or cytokines are possibilities to improve the effectiveness of CAR T-cells in lymphomas (NCT02650999; NCT00968760) [130,131].

## 5. Conclusions

Recent advances in our knowledge of the pathophysiology of BCL have allowed the development of new targeted therapies, combination treatments and immunotherapeutic approaches. Many of them have first been used in nodal BCL and these developments could benefit patients with CBCL. The identification of predictive biomarkers of response will help select the optimal therapeutic options. In the era of personalized medicine, large-scale translational studies and prospective clinical trials are mandatory to improve the management of these rare diseases.

## Figures and Tables

**Table 1:** Efficacy and safety of selected novel therapies in B-cell lymphomas.

Target	Treatment	Phase	Nb of pts	Pathology	ORR (%)	CR (%)	Median PFS (mo)	DOR (mo)	Median f-u (mo)	Adverse Effects	Reference
**Small Molecule Inhibitors**											
BTK	Ibrutinib	case report	1	PCDLBCL, LT	100	100	ND	18	ND	ND	Gupta et al.
BTK	Acalabrutinib	II	124	R/R MCL	81	40	NR	NR	15,2	headache, gd 3 hematologic events, pneumonia	Wang et al.
BTK	Zanubrutinib	I	78	R/R CLL/SLL	96.2	2.6	NR	ND	13.7	neutropenia, subcutaneous hemorrhage	Tam et al.
PI3Kδ	Parsaclisib	I/II	72	R/R NHL	20 to 100	0 to 44	ND	4.4 to 13.5	ND	diarrhea, nausea, neutropenia	Forero Torres et al.
PKCβ	Enzastaurin	III	758	DLBCL	80	ND	ND	ND	48	urine color change, prolonged QTc interval	Crump et al.
TLRs 7,8,9	IMO-8400	I/II	6	R/R DLBCL						Gastrointestinal disorders, hematologic events	NCT02252146
HDAC	Mocetinostat	II	72	R/R DLBCL, FL	18.9/11.5	2.7/3.8	2.1/3.7	ND	ND	fatigue, nausea, diarrhea	Batlevi et al.
Bcl-2	Venetoclax	I	106	R/R NHL	44	13	6	ND	ND	gd 3 hematologic events	Davids et al.
**Monoclonal Antibodies**											
CD20	Ofatumumab	III	36	FL	84	16	1.9	23.7	30.7	gd 3 infusion reactions	Rosenbaum et al.
CD20	Ofatumumab	II	11	R/R DLBCL	18	0	2	ND	38	diarrhea, anorexia, hyponatremia, fatigue	Galanina et al.
CD19 (+proteasome)	MOR-208 (+ lenalidomide)	II	81	R/R DLBCL	54	32	16,2	NR	12	gd 3 neutropenia	Salles et al.
CD40	Dacetuzumab	II	46	R/R DLBCL	9	4	1.2	ND	ND	thrombosis, ocular events, gd 3/4 hematologic events	Sven de Vos et al.
CD79 b (+ CD20)	Polatuzumab vedotin (+ rituximab)	II	59	R/R DLBCL, FL	54/70	21/45	5.6/15.3	13.4/9.4	17.4/NE	hematologic events, diarrhea	Morschhauser et al.
**BiTE Antibodies**											
CD19/CD3	Blinatumomab	II	21	R/R DLBCL	43	19	3.7	13.4	ND	tremor, gd 3 neurologic events	Viardot et al.
CD20/CD3	Mosunetuzumab	I/Ib	218	R/R NHL	43.8	25	ND	ND	ND	CRS 28.4%, neurological events 44%	Schuster et al.
**Fusion Proteins**											
CD20	DI-Leu16-IL2	I	9	NHL							NCT00720135
**Tumor Vaccine**											
Idiotype protein	Mitumprotimut T	III	174	FL	64	40	ND	ND	ND	injection site reactions	Freedman et al.
Idiotype gene	DNA vaccine	I	ND	NHL							ISRCTN31090206
APCs	Cellular vaccine	pilot	18	NHL	33.3	16%	ND	ND	50.5	injection site reactions	Di Nicola et al.
Dendritic cells	In situ vaccine	I/II	30	Indolent BCL							NCT01976585
**Immune Check Point Inhibitors**											
CTLA-4 (+ CD20)	Ipilimumab (+ rituximab)	I	33	R/R NHL	24	6	2.6	ND	ND	diarrhea, rash, abdominal pain	Tuscano et al.
**CAR T-Cell**											
CD19 CAR-T cells	Axicel	II	111	R/R LBCL	82	54	5.8	8.1	15.4	CRS 13%, neurological events 28%	Neelapu et al.
CD19 CAR-T cells	Tisagenlecleucel	II	93	R/R DLBCL	52	40	NR	ND	ND	CRS 22%, neurological events 12%	Schuster et al.
CD19 CAR-T cells (+ PD-1)	CD19 CAR-T cells (+ Nivolumab)		11	R/R DLBCL	81.8	45.4	6	6	6	CRS 50%, neurological events 1%	Cao et al.
CD19 CAR-T cells + PD-1	Pembrolizumab	I/II	12	R/R NHL after CD19 CAR-T cell							NCT02650999
CD19 CAR-T cells	CD19 CAR-T cells + IL-2	I	60	BCL							NCT00968760

APCs, antigen-presenting cells; BCL, B-cell lymphoma; BTK, Bruton tyrosine kinase; CLL/SLL, chronic lymphocytic leukemia/Small lymphocytic leukemia; CR, complete response; CRS, cytokine release syndrome; DLBCL diffuse large B-cell lymphoma; DOR, duration of response; f-u, follow-up; FL, follicular lymphoma; Gd, grade; HDAC, histone deacetylases; MCL, mantle cell lymphoma; mo, months; Nb, number; ND, not documented; NE, not estimable; NHL, non-Hodgkin lymphoma; NR, not reached; ORR, overall response rate; PCDLBCL, LT, primary cutaneous diffuse large B-cell lymphoma, leg type; PFS, progression free survival; PI3 Kδ, phosphatidylinositol 3-kinaseδ; PKCβ, protein kinase C β; Pts, patients; R/R, refractory/relapse.
